# Resveratrol suppresses IGF-1 induced human colon cancer cell proliferation and elevates apoptosis via suppression of IGF-1R/Wnt and activation of p53 signaling pathways

**DOI:** 10.1186/1471-2407-10-238

**Published:** 2010-05-26

**Authors:** Jairam Vanamala, Lavanya Reddivari, Sridhar Radhakrishnan, Chris Tarver

**Affiliations:** 1 Department of Food Science and Human Nutrition, 226 Gifford Building, Colorado State University, Fort Collins, CO, 80523-1571, USA; 2 Institute for Obesity Research and Program Evaluation, Texas A&M University, College Station, TX, 77843-2254, USA

## Abstract

**Background:**

Obesity is a global phenomenon and is associated with various types of cancer, including colon cancer. There is a growing interest for safe and effective bioactive compounds that suppress the risk for obesity-promoted colon cancer. Resveratrol (trans-3, 4', 5,-trihydroxystilbene), a stilbenoid found in the skin of red grapes and peanuts suppresses many types of cancers by regulating cell proliferation and apoptosis through a variety of mechanisms, however, resveratrol effects on obesity-promoted colon cancer are not clearly established.

**Methods:**

We investigated the anti-proliferative effects of resveratrol on HT-29 and SW480 human colon cancer cells in the presence and absence of insulin like growth factor-1 (IGF-1; elevated during obesity) and elucidated the mechanisms of action using IGF-1R siRNA in HT-29 cells which represents advanced colon carcinogenesis.

**Results:**

Resveratrol (100-150 μM) exhibited anti-proliferative properties in HT-29 cells even after IGF-1 exposure by arresting G_0_/G_1_-S phase cell cycle progression through p27 stimulation and cyclin D1 suppression. Treatment with resveratrol suppressed IGF-1R protein levels and concurrently attenuated the downstream Akt/Wnt signaling pathways that play a critical role in cell proliferation. Targeted suppression of IGF-1R using IGF-1R siRNA also affected these signaling pathways in a similar manner. Resveratrol treatment induced apoptosis by activating tumor suppressor p53 protein, whereas IGF-1R siRNA treatment did not affect apoptosis. Our data suggests that resveratrol not only suppresses cell proliferation by inhibiting IGF-1R and its downstream signaling pathways similar to that of IGF-1R siRNA but also enhances apoptosis via activation of the p53 pathway.

**Conclusions:**

For the first time, we report that resveratrol suppresses colon cancer cell proliferation and elevates apoptosis even in the presence of IGF-1 via suppression of IGF-1R/Akt/Wnt signaling pathways and activation of p53, suggesting its potential role as a chemotherapeutic agent.

## Background

Nearly two-thirds of Americans are overweight or obese [1], and obesity elevates the risk for colon cancer [2-9], the second leading cause of cancer deaths in the United States [10]. Surgery, radiation and chemotherapy alone or in combination are the most common methods to treat different types of cancers, including colon cancer. These conventional therapies can prolong a patient's life span, but they may cause serious side-effects. Since colon cancer takes 10-20 years to progress beyond initiation stage [11], there exists an opportunity to prevent colon cancer progression through appropriate nutrition and exercise. A recent meta-analysis found that a 5 kg/m^2 ^increase in body mass index (BMI) raises colon cancer risk by 24% in men [12]. Thus, it is imperative to intensify our efforts to better understand the pathogenesis of colon cancer during obese conditions and try to develop novel, evidence-based and safe approaches for its prevention and treatment.

The adipokines and growth factors are involved in a wide range of physiological processes including general homeostasis, cell growth, metabolism, and are frequently deregulated during obese conditions [13]. Over-activation of the IGF system is also commonly observed in obese conditions and plays a critical role in obesity-promoted colon cancer [14]. IGF-1 has been linked to tumor growth in adults in a number of studies, including two large trials jointly conducted by Harvard Medical School and Brigham and Women's Hospital. A six-year study of 32,826 nurses, found that those with the highest levels of IGF-1 had a two-and-a-half times greater risk of colorectal cancer; another study of 14,916 male physicians also concluded that men run the same risk [15,16]. This IGF system includes ligands, receptors, and ligand-binding proteins (IGFBPs). Over-nourishment and chronic hyperinsulinemia observed in obese conditions may deregulate colonocyte growth kinetics, as elevated insulin and suppressed IGFBP-1, and IGFBP-2 levels increase the pool of free or bioavailable IGF-1. A larger pool of bioavailable IGF-1 can activate the IGF-1 receptor (IGF-1R), thus may stimulate colonocyte proliferation [14,17-19]. IGF-1R is over expressed during colon carcinogenesis, with the highest expression in the proliferating cells at the base of the colonic crypts [20]. Upon IGF-1 binding, IGF-1R activates the PI3K/Akt cascade, which promotes G_1 _to S cell cycle progression [21] and elevates cell proliferation [22]. One of the pathways activated by the PI3K/Akt cascade is the Wnt/β-catenin pathway [23]. The Wnt/β-catenin pathway plays a central role in elevating colonocyte proliferation [24] and suppressing apoptosis in both humans and rodent models of experimentally induced colon cancer [25,26]. Akt phosphorylates and inactivates GSK3β, allowing β-catenin, a downstream effector of Wnt pathway to translocate into the nucleus where it binds to TCF-4. The β-catenin/TCF-4 complex constitutes the master switch that regulates colonocyte proliferation [27].

Resveratrol, a stilbenoid derived mainly from the skin of grapes, is a potential dietary compound against various cancers - breast, colon, etc., *in vitro *and *in vivo *[28,29]. Resveratrol works at a very low concentration *in vivo *(< 2 μM). However, *in vitro *studies require a much higher concentration (at least 25-50 μM) to elicit similar chemotherapeutic action [30]. In a variety of cancer cells, resveratrol suppressed critical components of the PI3K/Akt pathway [31-33]. Recent reports suggest that resveratrol also promotes phosphorylation of p53 in a dose and time-dependent manner in human breast cancer cells [34]. This impairs ability of p53 to bind to its negative regulator, MDM2 [35]. Thus p53 is active and can cause either cell cycle arrest or apoptosis [34,35]. P53 suppresses IGF-1R gene expression [36,37] in Saos-2 (osteosarcoma-derived cells) and RD (rhabdomyosarcoma-derived cells) cells [38]. Resveratrol also has been shown to suppress IGF-1 levels in mice consuming a high-fat diet [39]. Low concentrations of resveratrol (< 40 μM) have also shown to inhibit Wnt signaling in colon cancer cells [29]. However, the inhibitory efficacy of resveratrol against IGF-1 induced colonocyte proliferation and the molecular mechanisms of its action are not yet fully elucidated. We hypothesized that resveratrol suppresses colon cancer cell proliferation and elevates apoptosis after IGF-1 exposure through activation of p53 and suppression of IGF-1R/Akt/Wnt signaling pathways. In order to establish the efficacy of resveratrol against IGF-1 induced colon cancer growth, we utilized HT-29 and SW480 (IGF-1R constitutively active) human colon cancer cell lines and treated them with/without IGF-1 and/or resveratrol.

In this study, resveratrol at 100-150 μM suppressed IGF-1R/Akt and Wnt/β-catenin signaling pathway proteins in the HT-29 colon cancer cell lines. Similar results were observed with IGF-1R siRNA treatment. Resveratrol also activated p53 protein and suppressed levels of sp1, a protein that transcriptionally activates IGF-1R. Resveratrol elevated unphosphorylated (active) form of forkhead transcription factor (FKHRL1 protein), thereby contributing to cell cycle arrest. Overall our results demonstrate that resveratrol (i) suppresses IGF-1R levels, thus, downregulates Akt and Wnt/β-catenin signaling; (ii) elevates levels of active FKHRL1 and p27, and concomitantly suppresses cyclin D1 levels and (iii) activates p53 and suppresses sp1, thus suppressing cell cycle progression and elevating apoptosis *in vitro *even in the presence of free mitogenic IGF-1.

## Methods

### Chemicals

IGF-1 was obtained from the R&D Systems (Minneapolis, MN). Fetal bovine serum (FBS) was purchased from the Thermo Fisher Scientific (Pittsburgh, PA). All other cell culture supplies and resveratrol were purchased from the Sigma Chemical Company (St. Louis, MO).

### Cell lines

HT-29 and SW480 colon cancer cell lines were obtained from the American Type Culture Collection (Manassas, VA). Cells were maintained at 37°C with 5% CO_2 _and grown in Dulbecco's Modified Eagle's Medium F-12 (DMEM/F-12) supplemented with 2.2 g/L sodium bicarbonate, 0.2 g/L bovine serum albumin, 50 mL/L fetal bovine serum and 10 mL/L antibiotic and antimycotic solution.

### Design of siRNA against IGF-1R

IGF-1R siRNA and inverted control duplex were designed as described [40] and were purchased from Ambion (Austin, TX). The sequences of IGF-1R siRNA duplex are: sense strand, 5'-CGACUAUCAGCAGCUGAAGTT-3'; antisense strand, 5'-CUUCAGCUGCUGAUAGUCGTT-3'. It is homologous to 168-186 nucleotides of human IGF-1R transcript. 5'-GAAGUCGACGACUAUCAGCTT-3' and 5'-GCUGAUAGUCGUCGACUUCTT-3' are sense and antisense sequences of inverted control duplex, respectively. The construct against IGF-1R mRNA was referred as IGF-1R siRNA, the inverted control or non specific siRNA was referred as siRNA control, and GAPDH siRNA was used as positive control.

### siRNA transfection

HT-29 cells were seeded in DMEM/F-12 supplemented with 5% fetal bovine serum in six-well plates. Cells at 50% confluence were transfected with IGF-1R siRNA duplexes after 20-24 h, using Dharma *FECT *4 reagent (Dharmacon Inc., CO) in antibiotic- and serum-free DMEM/F-12 medium. Three concentrations (50, 75 and 100 nM) of IGF-1R siRNA duplexes, 100 nM non specific siRNA and 100 nM GAPDH siRNA were used for initial optimization, and for subsequent assays 75 nM siRNA was used, as there was no difference in IGF-1R inhibition between 75 and 100 nM concentrations. Cells were harvested after 72 h of transfection using 1× lysis buffer, and then protein was extracted. The knockdown of IGF-1R was confirmed by Western blotting.

### Cell proliferation assay

Cells were plated at a density of 5 × 10^4 ^cells per well in 12-well plates in Dulbecco's modified Eagle's medium F-12 (DMEM/F-12) containing 2.5% charcoal-stripped fetal bovine serum. After 24 h, cells were treated with DMSO (solvent control), 10 nM IGF-1 and/or resveratrol (150 μM), and anti-proliferative properties of resveratrol were evaluated after 24, 48 and 72 h. As colonocytes have greater exposure to the bioavailable IGF-1 in obese condition, in the combined treatments (for all the studies in the manuscript), the cells were pre-incubated with IGF-1 (10 nM) for 5 minutes followed by resveratrol treatment to check the effect of resveratrol when the cells are already primed to proliferate. Dose response studies with IGF-1 (5 - 20 nM) showed that 10 and 20 nM IGF-1 treatments did not differ (p < 0.05) in inducing cell proliferation (data not shown). Thus, we used 10 nM concentration of IGF-1 for subsequent experiments. In case of siRNA experiment, after 24 h of transfection, cells were treated with IGF-1 at 10 nM and/or resveratrol at 150 μM for 24 h. Cells were counted after 24 and 48 h using a Z1 Coulter Counter, Beckman Coulter (Fullerton, CA). Each experiment was carried out in triplicate, and results were expressed as mean ± SE.

### TUNEL assay

The TUNEL (terminal transferase dUTP nick end labeling) assay was performed to determine the effect of resveratrol/IGF-1R siRNA on apoptosis. Cells (4 × 10^4^) were seeded in four-chambered glass slides and after treatment for 12 h, the *in situ *cell death detection kit, POD (peroxidase), Roche Applied Science (Indianapolis, IN) was used according to the instruction manual protocol for fixed cells to identify apoptotic cells. Slides incubated without terminal deoxynucleotidyl transferase (TdT) served as a negative control, and slides treated with 1000 U DNAse I/ml for 10 min before TdT exposure served as a positive control. After the incubation of cells with POD and diaminobenzidene substrate, the percentage of apoptotic cells was calculated by counting the stained cells in 12 fields, each containing at least 50 cells [41].

### Fluorescence-activated cell sorting analysis (FACS)

HT-29 cells were plated at a density of 15 × 10^5 ^cells per 100 mm plate and after 18 h treatment with either the control (DMSO), IGF-1 (10 nM), resveratrol (50 or 150 μM), or their combinations, cells were trypsinized and centrifuged. The pellet was resuspended with 1 ml of PI staining buffer containing 4 mM sodium citrate, 0.1% Triton X-100, 50 μg/ml propidium iodide and 200 μg/ml RNAse and incubated for 10 min at 37°C in the dark, and the final concentration of sodium chloride was adjusted to 0.15 M. Cells were analyzed using FACSCalibur flow cytometer and CellQuest Acquisition software, Becton Dickinson immunocytometry systems (San Jose, CA). Results were reported as per cent cells in each phase of the cell cycle.

### Western blot analysis

HT-29 cells were seeded at a density of 1.5 × 10^5 ^cells/mL in Dulbecco's Modified Eagle's Medium F-12 with 2.5% charcoal-stripped fetal bovine serum for 24 h. Cells were treated with control, IGF-1, different concentrations of resveratrol (50, 100 and 150 μM) with and without IGF-1 (10 nM) for 24 h. Protein was extracted into a high-salt buffer containing 1% protease inhibitor cocktail from Sigma-Aldrich (St. Louis, MO), and protein concentrations were determined by a BCA Protein Assay kit from Pierce (Rockford, IL). Cell lysates (30 μg) were incubated at 98°C for 5 min and separated by Novex^® ^8-16% Tris-HCl gels from Invitrogen (Carlsbad, CA) at 120 V for 2 h in 1 × running buffer [25 mmol/L Tris, 192 mmol/L glycine, 0.1% SDS (pH 8.3)], and electrophoretically transferred to Immun-Blot PVDF membranes from Bio-Rad Laboratories (Hercules, CA) at 95 V for 35 min in 2× Tris-glycine transfer buffer (Novex, LC3675, Invitrogen) with 0.025% SDS. PVDF membranes were blocked with 2% bovine serum albumin from Fisher Scientific (Pittsburg, PA) for 1 h at room temperature. The membranes were incubated with rabbit polyclonal anti-sp1 antibody, Upstate Biotechnology (1:1,000; Lake Placid, NY), goat polyclonal anti-β-catenin antibody, rabbit polyclonal anti-GSK3β antibody, mouse monoclonal anti-cyclin D1 antibody, goat polyclonal anti-β-actin antibody, mouse monoclonal anti-MDM2 antibody, rabbit polyclonal anti-p27 antibody, rabbit polyclonal anti-IGF-1Rβ antibody and rabbit polyclonal anti-FKHRL1 antibody, all from Santa Cruz Biotechnology (1:500; Santa Cruz, CA) for 2 h at room temperature. Membranes were subsequently probed with bovine anti-goat, goat anti-rabbit or goat anti-mouse IgG-HRP conjugate secondary antibody, Santa Cruz Biotechnology (all 1:100,000). Target proteins were detected with Super Signal West Dura Extended Duration Substrate from Pierce (Rockford, IL). Membranes were scanned with ChemiDoc XRS (Bio-Rad) using Quantity One software (Bio-Rad) and the band intensities were calculated using Image J software, and normalized to β-actin, a loading control.

### Statistical analysis

Analysis of variance and Fisher least square difference at 5% significance level determined the degree of significance among treatments. The results were expressed as mean ± SE for three replicates for each treatment.

## Results

### Growth stimulatory/inhibitory effects of IGF-1 and resveratrol

**Figure 1:**
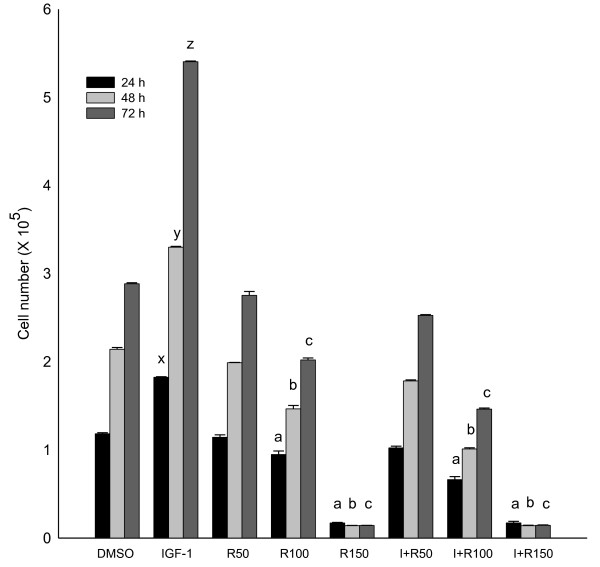
**Growth stimulatory and inhibitory effects of IGF-1 and resveratrol, respectively in HT-29 colon cancer cells**. HT-29 cells were treated with IGF-1 (I; 10 nM) and/or resveratrol (R; 50, 100, and 150 μM) for 24, 48 and 72 h, and cell numbers were determined using a Z1 Coulter counter. Results were expressed as mean ± SE for three experiments at each time point. Mean proliferation (p < 0.05) lower than that of control treatment are indicated: a, b and c for 24, 48 and 72 h, respectively and higher (p < 0.05) than that of control are indicated: x, y and z for 24, 48 and 72 h, respectively.

**Figure 2:**
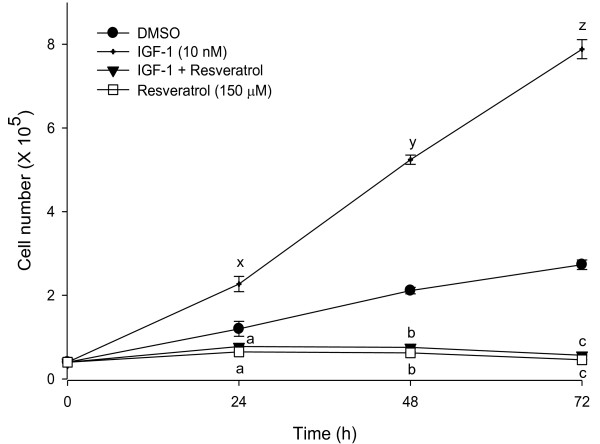
**IGF-1 elevates and resveratrol suppresses cell proliferation in SW480 colon cancer cells**. SW480 cells were treated with IGF-1 (10 nM) and/or resveratrol (150 μM) for 24, 48 and 72 h and cell numbers were determined using a Z1 Coulter counter. Results were expressed as mean ± SE for three experiments at each time point. Mean proliferation significantly lower than that of control treatment are indicated: a, b and c for 24, 48 and 72 h, respectively (p < 0.05) and higher than that of control are indicated: x, y and z for 24, 48 and 72 h, respectively (p < 0.05).

Growth stimulatory effects of IGF-1 and the anti-proliferative effects of resveratrol were investigated using HT-29 and SW-480 colon cancer cells. Human colon cancer cells were grown in DMEM with 2.5% charcoal-stripped serum for 24 h and treated with DMSO (solvent control), IGF-1 (10 nM), and different concentrations of resveratrol (50, 100 and 150 μM). Our results demonstrated that resveratrol suppresses the colon cancer cell proliferation even when the cells are primed to proliferate with IGF-1. After 72 hours, IGF-1 (10 nM) treatment increased HT-29 cell proliferation (87%) confirming its growth stimulatory effects (Figure 1). Treatment of these cells with resveratrol suppressed cell proliferation (up to 95%) compared to the control. Moreover, pre-incubation of HT-29 cells with IGF-1 for 5 minutes followed by treatment with resveratrol also resulted in suppressed cell proliferation (up to 94%) (Figure 1). The colon cancer cell number in the combination of IGF-1 and resveratrol treatment was similar to resveratrol treatment alone. Thus, resveratrol might be a potent inhibitor of cell proliferation even in the presence of IGF-1. Similar results were obtained using SW-480 colon cancer cells (Figure 2). Resveratrol suppresses cell proliferation to levels below control even in the presence of IGF-1. This may be due to resveratrol's combined effect on IGF-1R signaling suppression, induction of apoptosis and alteration of multiple other pathways. Cytotoxicity assays established that resveratrol did not induce necrosis at the doses used for this study (data not shown). Since HT-29 colon cancer cells represent advanced stages of colorectal cancer, further experiments were conducted using HT-29 cells [42,43].

**Figure 3:**
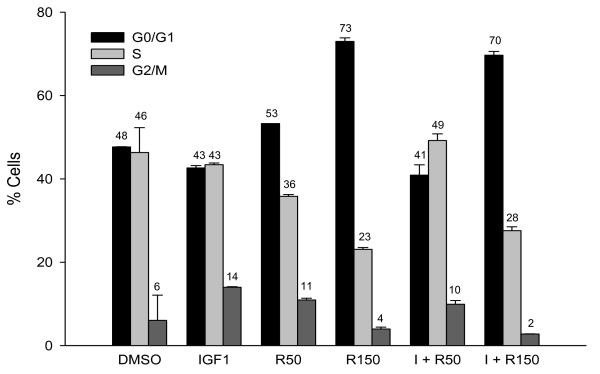
**Effect of resveratrol on cell cycle progression of IGF-1 treated colon cancer cells**. HT-29 cells were plated at a density of 15 × 10^5 ^cells per 100 mm plate and after 18 h treatment with the vehicle (DMSO), IGF1 (I; 10 nM) and/or resveratrol (R; 50 or 150 μM); they were analyzed by Fluorescence-activated cell sorting analysis (FACS). Results are expressed as % of cells in each phase. Numbers on top of the bars represent percent cells in respective phases in each treatment group.

Fluorescence-activated cell sorting analysis (FACS) technique was used to determine the effect of resveratrol on cell cycle progression in HT-29 cells. Figure 3 illustrates the distribution of HT-29 cells in the G_0_/G_1_, S and G_2_/M phases after IGF-1 (10 nM) and/or resveratrol (50 μM or 150 μM) treatments. IGF-1 treatment had lower percentage of HT-29 cells in the G_0_/G_1 _phase (42.6%), whereas resveratrol elevated the percentage of HT-29 cells in the G_0_/G_1 _phase (72.9%), compared to the control. Combined treatments of IGF-1 and resveratrol also caused an increase in the percentage of cells in the G_0_/G_1 _phase (69.7%) similar to resveratrol treatment alone. Resveratrol treatment at 50 μM also had numerical increase in cells in G_0_/G_1 _phase, however it was not significantly different from the control. These results suggest that resveratrol targets G_0_/G_1 _to S phase progression to arrest IGF-1 induced HT-29 cell proliferation.

### Efficacy of IGF-1R siRNA in suppressing IGF-1 stimulated growth of colon cancer cells

**Figure 4:**
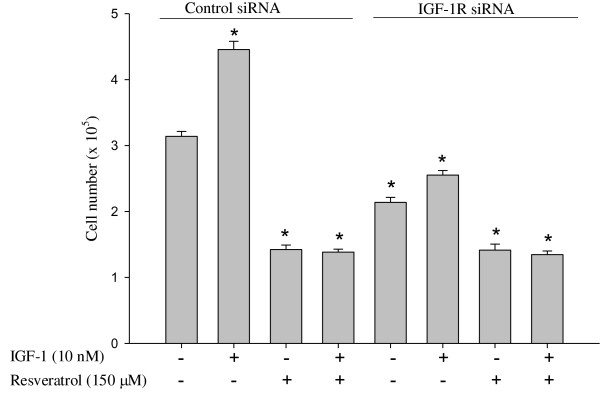
**Effect of IGF-1R knockdown and/or resveratrol on proliferation of colon cancer cells *in vitro***. Cells were seeded in 12-well plates and transfected with IGF-1R siRNA duplexes (75 nM) or non specific siRNA (siRNA control). After 24 h of transfection, cells were treated with IGF-1 (10 nM) and/or resveratrol (150 μM) for 24 h. Cell numbers were determined using a Coulter counter. * indicates suppression or elevation (p < 0.05) by treatments compared to non specific siRNA control treatment.

To determine whether the IGF-1 induced growth stimulatory properties are through IGF-1R, HT-29 cells were transfected with either non specific siRNA (siRNA control) or IGF-1R siRNA (75 nM) and after 24 h treated with DMSO (solvent control), IGF-1 (10 nM) and/or resveratrol (150 μM). IGF-1R siRNA suppressed colon cancer cell proliferation, and the cell numbers were lower than the siRNA control group (Figure 4). IGF-1 treatment could not increase the number of cells in IGF-1R siRNA transfected group, but it did increase (p < 0.05) the cell proliferation in non specific siRNA transfected cells compared to the solvent control, confirming that IGF-1 induced proliferation is through IGF-1R. IGF-1R siRNA along with resveratrol suppressed proliferation compared to IGF-1R siRNA alone. The effect of resveratrol on IGF-1R is similar to the inhibitory effects of IGF-1R siRNA on the proliferation of colon cancer cells via suppressing IGF-1R and the downstream kinases, and the Wnt/β-catenin signaling pathway.

### Resveratrol but not IGF-1R siRNA induces apoptosis in HT-29 cells

**Figure 5:**
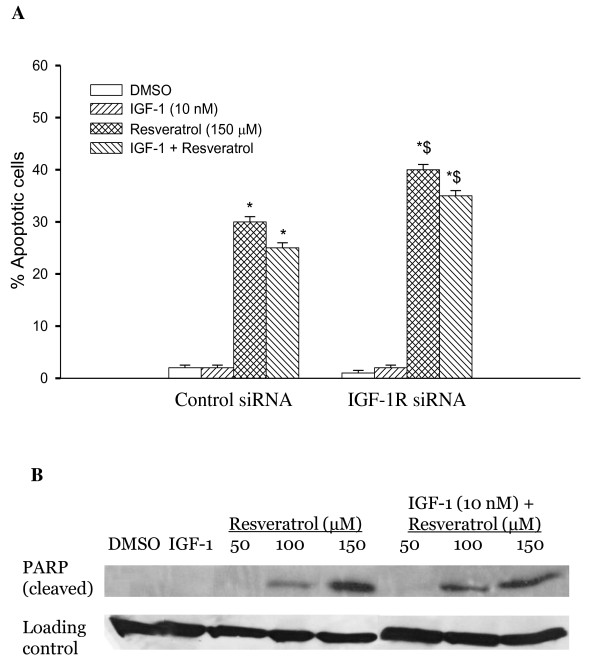
**Effects of Resveratrol and treatment on apoptosis**. **A - Resveratrol but not IGF-1R siRNA induces apoptosis in HT-29 cells**. Percentage induction of apoptosis was determined by TUNEL assay. The rate of apoptosis was expressed as a percentage of the total cells counted. Results are expressed as mean ± SE for the different treatments in both non specific siRNA and IGF-1R siRNA transfected cells. * indicates significant elevation compared to solvent control (DMSO). ^$^indicates elevation (p < 0.05) by treatments compared to non specific siRNA control. **B - Resveratrol treatment elevated cleaved PARP, a hallmark of apoptosis**. Cells were treated with resveratrol (50, 100 and 150 μM) alone or IGF-1 (10 nM) for 5 minutes followed by resveratrol at different concentrations and analyzed by western blot as described in materials and methods. Similar results were obtained in duplicate experiments.

Induction of apoptosis by resveratrol and/or IGF-1R siRNA in HT-29 cells was analyzed using TUNEL (terminal transferase dUTP nick end labeling) assay. Cells were grown in four chambered glass slides and transfected with either non specific siRNA (siRNA control) or IGF-1R siRNA and treated with IGF-1 and/or resveratrol. IGF-1R siRNA did not alter apoptosis suggesting that IGF-1R siRNA suppresses colon cancer cell growth primarily through anti-proliferative and/or senescence mechanisms. Resveratrol induced 30% and 25% of apoptotic cell death in the absence and presence of IGF-1, respectively, in the siRNA control group (Figure 5A). These results suggest that unlike IGF-1R siRNA, resveratrol inhibits growth of colon cancer cells by not only suppressing proliferation but also elevating apoptosis. In the case of IGF-1R siRNA transfected cells, the percentage induction of apoptosis with resveratrol, alone or in the presence of IGF-1, was about 40% and 30%, respectively (p < 0.05, Figure 5A). To further confirm the apoptotic cell death by resveratrol, we measured PARP cleavage, the hallmark of apoptosis, using Western blotting. Figure 5B shows the induction of PARP cleavage as accumulation of cleavage fragment (89 kDa) in resveratrol treated HT-29 cells.

### Resveratrol down regulates IGF-1R signaling proteins

**Figure 6:**
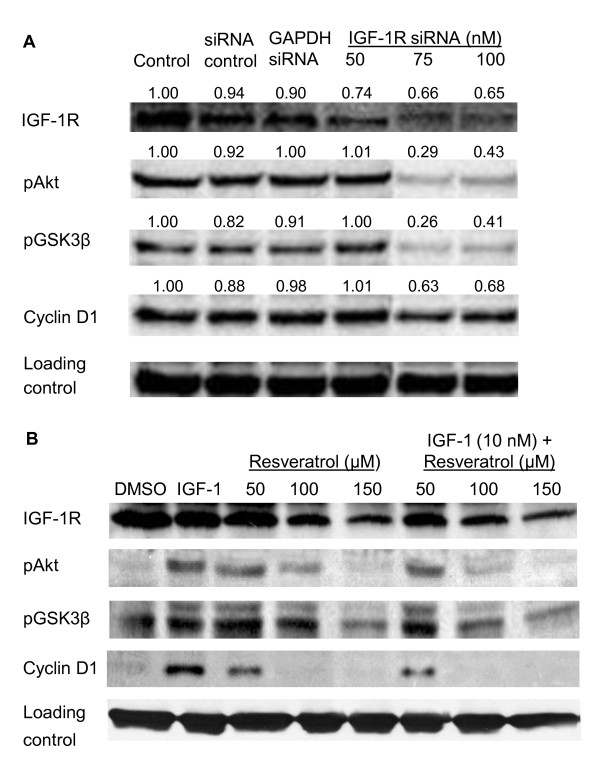
**Effect of IGF-1R siRNA (A), and resveratrol (B) on IGF-1R/Wnt signaling pathway proteins**. **A **- HT-29 cells were treated with DMSO (control), non specific siRNA (siRNA control; 100 nM), positive siRNA (GAPDH siRNA; 100 nM) and IGF-1R siRNA (50, 75 and 100 nM) for 72 h. **B **- HT-29 cells were treated with DMSO, IGF-1 (10 nM) and/or resveratrol (150 μM) for 24 h. In case of both A and B, whole cell lysates were analyzed by western blotting as described in materials and methods. Proteins were detected by using specific antibodies. Band intensities normalized to loading control were indicated on top of the respective band (A). Similar results were obtained in duplicate experiments.

**Figure 7:**
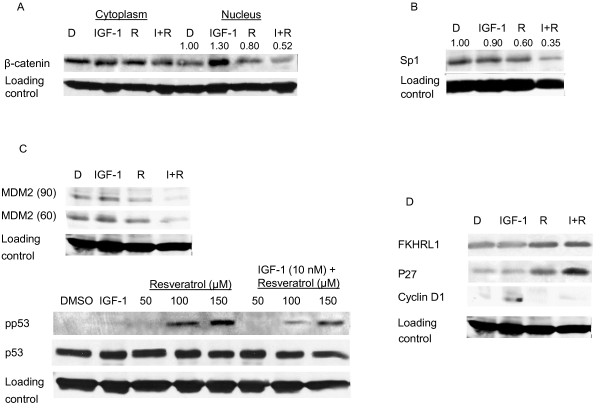
**Suppressed human colon cancer cell proliferation and elevated apoptosis by resveratrol involves multiple molecular targets**. Effects of resveratrol (R; 150 μM) and/or IGF-1 (I; 10 nM) on expression of **A**, β-catenin; **B**, sp1; **C**, MDM2 and p53; **D**, FKHRL1, p27 and cyclin D1; in HT-29 cells. HT-29 cells were treated with IGF-1 and/or resveratrol for 24 h. Whole cell lysates were analyzed by western blotting as described in materials and methods. Blots were incubated with the indicated antibodies. Band intensities normalized to loading control were indicated on top of the respective band (A, B). Similar results were obtained in duplicate experiments.

To investigate whether resveratrol/IGF-1R siRNA induced growth arrest of the human colon cancer cell lines was associated with IGF-1R downstream signaling, lysates from cells treated with IGF-1, resveratrol, and IGF-1R siRNA were subjected to western blot analysis. IGF-1 is a known cell cycle progression factor that activates important downstream kinases, PI3K/Akt. IGF-1 treatment elevated pAkt, pGSK3β, and cyclin D1 levels resulting in augmented proliferation of HT-29 colon cancer cells (Figure 6B). IGF-1R knockdown using siRNA suppressed the levels of IGF-1R protein and concomitantly suppressed the downstream kinases, pAkt and pGSK3β, and cyclin D1 (Figure 6A). Similar results were observed with resveratrol alone or in combination with IGF-1 (Figure 6B). Cyclin D1 is an important protein for cell cycle progression and its levels are dependent on β-catenin nuclear translocation [44]. Figure 7A shows lower levels of nuclear β-catenin in resveratrol treated cells compared to control or IGF-1 treated cells. Interestingly, IGF-1 pretreatment potentiated the resveratrol suppression of nuclear β-catenin levels.

Sp1 is a critical mediator of IGF-1R transcription and upregulates IGF-1R promoter activity [38,45]. Figure 7B illustrates the effect of IGF-1 and resveratrol on sp1 protein levels. Resveratrol downregulated sp1 protein and suppressed the levels of IGF-1R (Figure 7B, 6B). These results suggest that IGF-1R is an important molecular target for resveratrol in the suppression of colon cancer cell proliferation and also suggests the role of resveratrol in regulating IGF-1R transcription. To elucidate whether or not resveratrol affects the insulin receptor (IR), we determined IR protein levels. We did not observe down regulation of IR receptor, even with a high concentration of resveratrol (data not shown), which confirms selective targeting of IGF-1R by resveratrol.

### Resveratrol activates tumor suppressor protein p53 and elevates activated forkhead transcription factor 1 levels

A critical tumor suppressor gene p53 regulates DNA repair, cell cycle progression and apoptosis [46]. In cancer cells, p53 is generally degraded due to ubiquitination by MDM2, a negative regulator of the p53 [46]. Our results show that resveratrol activates p53 and suppresses MDM2 levels in colon cancer cells (Figure 7C). The observed reduction in IGF-1R protein levels in resveratrol treatment may be in part due to p53 activation, because p53 is involved in transcriptional suppression of the IGF-1R gene, and regulation of the IGF-1R promoter activity through suppression of sp1 [38,45].

FKHRL1, a member of the family of forkhead transcription factors, is a transcriptional activator that induces cell cycle arrest or upregulates apoptosis depending upon cellular context and type of stress [47]. Unphosphorylated FKHRL1 was upregulated in cells treated with resveratrol (Figure 7D). P27, a downstream target of FKHRL1, is a cell cycle regulator protein that binds to and inactivates cyclin D1 [47]. P27 was also upregulated by resveratrol treatment (Figure 7D). Combined, these results suggest that resveratrol may cause cell cycle arrest at the G_0_/G_1_-S phase transition via FKHRL1 pathway by upregulating p27 and inactivating cyclin D1.

## Discussion

Phytochemicals affect myriad intracellular targets; it is this quality that often makes them desirable as chemotherapeutic agents against cancer [17,41,48-50]. Studies in both *in vitro *and *in vivo *models suggest that resveratrol can suppress components of the IGF system [32,33], which in turn may suppress cell proliferation and elevate apoptosis. Our results indicate that resveratrol suppresses cell proliferation and induces apoptosis even when the cells are primed to proliferate with IGF-1, a mitogen that is highly bioavailable during obesity. Resveratrol exerts its anti-proliferative and pro-apoptotic properties through suppression of IGF-1R/Akt/Wnt pathways and activation of p53 signaling.

Emerging evidence suggests that members of the insulin-like growth factors (IGFs) family, including IGF-I, IGF-II, IGF-IR, and the IGF-binding proteins (IGFBPs) play a central role in the development and progression of a variety of cancers during obesity, including colon cancer [51]. IGF-1 binding to IGF-1R stimulates downstream proliferating pathways such as the PI3K/Akt [52] and Ras signaling [23] resulting in increased human colon cancer cell proliferation, thus suppressing IGF-1R might attenuate proliferation. Resveratrol suppressed IGF-1 stimulated HT-29 colon cancer cell proliferation. Pre-incubation with IGF-1 followed by resveratrol suppressed proliferation lower than that of control and was similar to that of treatment with resveratrol alone. This may be due to resveratrol's combined effect on IGF-1R signaling suppression, apoptosis and multiple other pathways. Similar results were also observed in SW480 cells with constitutively expressed IGF-1R. Indeed, FACS results show that treatment with resveratrol alone or in combination with IGF-1 arrests G_0_/G_1_-S phase progression. These results indicate that resveratrol may suppress cellular proliferation even when colon cancer cells are already exposed to high levels of IGF-1 during obese conditions.

Knockdown of IGF-1R using IGF-1R siRNA also suppressed cell proliferation comparable to resveratrol treatment even in the presence of IGF-1. Resveratrol induced apoptosis in HT-29 cells, but IGF-1R siRNA had no effect on apoptosis. However, resveratrol treatment had greater percent apoptosis in IGF-1R siRNA transfected cells compared to non specific siRNA. Whether provided alone or following incubation with IGF-1, resveratrol suppressed the IGF-1R/Akt/GSK3β signaling pathway and concomitantly suppressed nuclear translocation of β-catenin. Once inside the nucleus, β-catenin binds to TCF-4 and transcriptionally activates proliferative (e.g., cyclin D1) genes. Cyclin D1 is known to function as a regulatory subunit of CDK4 or CDK6 (cyclin dependant kinases), whose activity is required for G_0_/G_1_-S phase transition [53]. Interestingly, IGF-1 pretreatment potentiated the resveratrol suppression of nuclear β-catenin. This may be because resveratrol targets actively proliferating cells and IGF-1 promotes number of proliferating cells via G1 to S cell cycle progression. This is in accordance with greater resveratrol suppression of proliferation in IGF-1 pretreated cells. IGF-1R siRNA transfection also affected the downstream Akt/GSK3β/β-catenin pathway similar to that of resveratrol. These results suggest that resveratrol exerts its anti-proliferative properties through suppression of IGF-1R, and is similar to IGF-1R siRNA in suppressing HT-29 cell proliferation but not in the induction of apoptosis.

In normal, unstressed cells, p53 levels are low due to continuous MDM2-mediated ubiquitination and degradation. IGF-1R activation further ensures low levels of p53 as the resulting increase in phosphorylated Akt increases the ubiquitination activity of MDM2 [46]. However, p53 phosphorylation prevents p53 from binding to its negative regulator MDM2 [35]. Reports suggest that p53 activation suppresses the IGF-1R mRNA levels [36]. The observed reduction in IGF-1R protein levels in resveratrol treatment may be in part due to p53 activation, because p53 is involved in transcriptional suppression of the IGF-1R gene, and regulation of the IGF-1R promoter activity through suppression of sp1 [38,45]. Resveratrol treatment exerted anti-proliferative effects without suppressing IR levels (data not shown), suggesting that resveratrol specifically targets IGF-1R in suppressing cell proliferation by activating p53 and suppressing sp1 levels.

Transcriptional activation of FKHRL1 proteins is regulated by the serine/threonine kinase Akt, which phosphorylates FKHRL1 and inactivates it [54,55]. Induction of apoptosis or withdrawal of growth factors stimulates dephosphorylation and nuclear translocation of FKHRL1, leading to FKHRL1-induced gene-specific transcriptional activation [54,56-60]. We studied the effect of resveratrol on FKHRL1. Resveratrol caused up-regulation of unphosphorylated active form of FKHRL1. This active form of FKHRL1 translocates to the nucleus and has been shown to induce apoptosis by upregulation of Fas-ligand expression and activation of the death receptor pathway [61]. P27 is a direct downstream target of FKHRL1 and over expression of cytoplasmic FKHRL1 concomitantly elevates p27, an inhibitor of cyclin D1, thereby affecting cell cycle progression [47,61]. Our data suggests that resveratrol up-regulates FKHRL1 in the cytoplasm and also increases p27 levels. Over expression of FKHRL1 resulted in an increased expression of p27 and thus potential inhibition of cyclin D1, and concurrent suppression of cell proliferation and cell cycle arrest as observed in this study.

However, resveratrol at lower concentrations (upto 50 μM) is not sufficient to elicit its chemotherapeutic properties in HT-29 cells. A lower dose of resveratrol is not strong enough to induce p53 activation (Figure 7C). As a result, the cancer cells upregulate the survival machinery through activation of survival pathways such as Akt/GSK3β/Wnt signaling at low doses of resveratrol (Figure 6B). However, at lower doses of resveratrol (50 μM), there is no significant effect on proliferation of HT-29 cells compared to control (Figure 1). At higher concentrations (100-150 μM), there is an elevation of activated p53 levels and suppression of IGF-1R/Akt/Wnt signaling pathways leading to suppressed cell proliferation, and enhanced apoptosis. Several studies indicate that for *in vitro *studies, carcinogenesis-modulating effects of resveratrol require the sustained presence of at least 5 to 100 μM [28,30]. Even though several preclinical efficacy studies [28,62,63] on resveratrol have reported that very low plasma concentrations of resveratrol (20 nM - 2 μM) are sufficient to exert potent cancer chemotherapeutic efficacy and pharmacodynamic activity, such *in vivo *levels would be insufficient to induce anti-carcinogenic effects in *in vitro *studies. Detailed studies need to be conducted in order to determine the dose difference in resveratrol's anti-proliferative and pro-apoptotic effects observed *in vivo *vs. *in vitro*.

**Figure 8:**
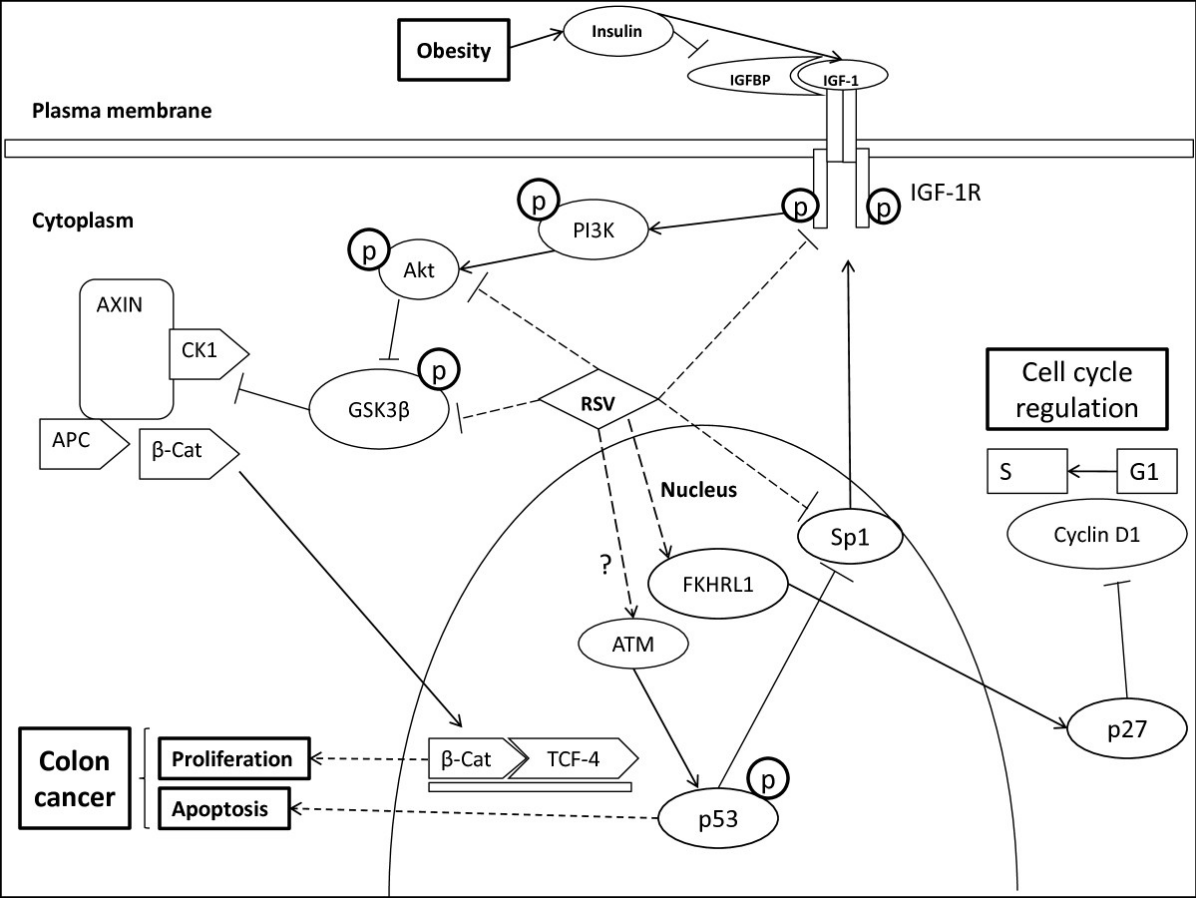
**Schematic diagram showing possible mechanisms by which resveratrol (RSV) alters colon cancer cell kinetics**. Obesity enhances insulin levels, which in turn elevates "bioactive IGF-1" levels by suppressing inhibitory IGF binding proteins (IGFBPs)-1 and -2. Activated IGF-1R phosphorylates a cascade of kinases PI3K, Akt and GSK3β. Phosphorylated and inactive GSK3β fails to form Axin/adenomatous polyposis coli (APC)/casein kinase 1 (CK1)/GSK3β destruction complex resulting in stabilization of cytosolic β-catenin and its subsequent nuclear translocation [reviewed in [64]]. In the nucleus, β-catenin binds to T-cell-factor 4 (TCF4) and stimulates the transcription of proliferative (e.g., cyclin D1) genes. The tumor suppressor p53, suppresses IGF-1R gene expression and phosphorylation. P53 attenuates IGF-1R transcription through its suppression of sp1, a critical mediator of IGF-1R transcription. Unphosphorylated, active FKHRL1 activates downstream target p27 and thus inhibiting cyclin D1 levels essential for cell cycle progression from G1 to S phase. Overall resveratrol suppresses IGF-1 induced colon cancer cell proliferation by A) downregulating IGF-1R levels, B) suppressing downstream IGF-1R/Akt and Wnt/β-catenin signaling C) activating p53 tumor suppressor protein, and by D) activation of FKHRL1/p27 signaling pathways.

Overall we have shown that resveratrol (i) mainly targets IGF-1R to suppress IGF-1R/Akt and Wnt/β-catenin signaling; but does not affect IR (insulin receptor) levels, thereby works similar to IGF-1R siRNA in suppression of proliferation but not in the induction of apoptosis (ii) elevates levels of active FKHRL1 and p27, and concomitantly suppresses cyclin D1 levels and (iii) activates p53 and suppresses sp1, thereby affecting IGF-1R transcription; thus suppressing cell cycle progression and elevating apoptosis *in vitro *even in the presence of free mitogenic IGF-1 (Figure 8).

## Conclusion

In conclusion, we have shown resveratrol to suppress IGF-1 (IGF-1 levels are elevated during obesity) induced cell proliferation and elevate apoptosis in human colon cancer cells, and elucidated the mechanisms of action using IGF-1R siRNA (Figure 8). Results of the current study will improve our understanding of the molecular pathways involved in the chemotherapeutic intervention of IGF-1 promoted colon tumorigenesis by the bioactive compound resveratrol. Understanding how IGF-1, Wnt/β-catenin and p53 pathways regulate colon cancer cell kinetics may help in the development of new diagnostic markers, prognostic markers, therapeutic targets and/or evidence based safe chemotherapeutic strategies against IGF-1 promoted cancers. We are working on detailed mechanistic studies and generating *in vivo *data to further decipher the underlying mechanism of the connection between obesity and colon cancer.

## Abbreviations

IGF-1: Insulin like growth factor 1; TCF-4: T cell transcription factor 4; MDM2: Murine double minute 2; IGF-1R: Insulin like growth factor 1 receptor; FKHRL1: Forkhead (Drosophila) homolog (rhabdomyosarcoma) like 1; PI3K: Phosphoinositide 3 kinase; Akt: Protein kinase B; GSK3β: Glycogen synthase kinase 3 beta.

## Competing interests

The authors declare that they have no competing interests.

## Authors' contributions

JV, LR and CT performed experiments and drafted the paper. SR ran some of the western blots and worked on the final version of the manuscript. JV conceived the study, participated in its design and coordination, and corrected the final version of the manuscript. All authors read and approved the final manuscript.

## Pre-publication history

The pre-publication history for this paper can be accessed here:

http://www.biomedcentral.com/1471-2407/10/238/prepub
